# Gonadal mosaicism of large terminal de novo duplication and deletion in siblings with variable intellectual disability phenotypes

**DOI:** 10.1002/mgg3.954

**Published:** 2019-09-01

**Authors:** Muhammad M. Rahman, KM Furkan Uddin, Nesreen K. Al Jezawi, Noushad Karuvantevida, Hosneara Akter, Nushrat J. Dity, Md. Ashiquir Rahaman, Maksuda Begum, Md. Atikur Rahaman, Md. Abdul Baqui, Zeena Salwa, Serajul Islam, Marc Woodbury‐Smith, Mohammed Basiruzzaman, Mohammed Uddin

**Affiliations:** ^1^ Bangabandhu Sheikh Mujib Medical University Dhaka Bangladesh; ^2^ NeuroGen Technologies Ltd. Dhaka Bangladesh; ^3^ Holy Family Red Crescent Medical College Dhaka Bangladesh; ^4^ College of Medicine Mohammed Bin Rashid University of Medicine and Health Sciences Dubai United Arab Emirates; ^5^ Department of Biotechnology Bharathidasan University Tiruchirappalli India; ^6^ Square Hospital Ltd. Dhaka Bangladesh; ^7^ IBN Sina Medical Imaging Center Dhaka Bangladesh; ^8^ Institute of Neuroscience Newcastle University Newcastle upon Tyne UK; ^9^ The Centre for Applied Genomics, Department of Genetics and Genome Biology The Hospital for Sick Children Toronto ON Canada; ^10^ Department of Neurology Dhaka Medical College Hospital Dhaka Bangladesh

**Keywords:** copy number variation, gonadal mosaicism, intellectual disability

## Abstract

**Background:**

Intellectual disability (ID) is a complex condition that can impact multiple domains of development. The genetic contribution to ID’s etiology is significant, with more than 100 implicated genes and loci currently identified. The majority of such variants are rare and de novo genetic mutations.

**Methods:**

We have applied whole‐genome microarray to identify large, rare, clinically relevant copy number variants (CNVs). We have applied well‐established algorithms for variants call. Quantitative polymerase chain reaction (qPCR) was applied to validate the variants using three technical replicates for each family member. To assess whether the copy number variation was due to balanced translocation or mosaicism, we further conducted droplet digital PCR (ddPCR) on the whole family. We have, as well, applied “critical‐exon” mapping, human developmental brain transcriptome, and a database of known associated neurodevelopmental disorder variants to identify candidate genes.

**Results:**

Here we present two siblings who are both impacted by a large terminal duplication and a deletion. Whole‐genome microarray revealed an 18.82 megabase (MB) duplication at terminal locus (7q34‐q36.3) of chromosome 7 and a 3.90 MB deletion impacting the terminal locus (15q26.3) of chromosome 15. qPCR and ddPCR experiments confirmed the de novo origin of the variants and the co‐occurrence of these two de novo events among the siblings, but their absence in both parents, implicates an unbalanced translocation that could have mal‐segregated among the siblings or a possible germline mosaicism. These terminal events impact *IGF1R, CNTNAP2,* and *DPP6*, shown to be strongly associated with neurodevelopmental disorders. Detailed clinical examination of the siblings revealed the presence of both shared and distinct phenotypic features.

**Conclusions:**

This study identified two large rare terminal de novo events impacting two siblings. Further phenotypic investigation highlights that even in the presence of identical large high penetrant variants, spectrum of clinical features can be different between the siblings.

## 1 INTRODUCTION

Intellectual disability (ID) is a severe, lifelong neurodevelopmental disorder that impacts intellectual capacity and adaptive functioning. According to the National Survey of Children's Health (NSCH), ID affects about 1.2% of children with a spectrum of severity ranging from profound to mild deficits (Maenner et al., 2016). Individuals with ID have a higher rate of earlier mortality than the population‐at‐large.

ID often co‐occurs with other neurodevelopmental disorders, such as autism spectrum disorder (ASD), attention deficit hyperactivity disorder, and epilepsy, and psychiatric disorders, such as anxiety, depression, and psychosis (Singh, Singh, Sahu, & Tikka, 2018). The prevalence of these mental comorbidities among the individuals with ID is as high as 40%. Indeed, a systematic review found that 30%–50% and 8%–18% of children and adolescents with and without an ID, respectively, have a mental disorder. Physical health conditions are also common: for example, gastroesophageal reflux is seen among 50% with ID. Craniofacial dysmorphism may also be observed among individuals with ID, such as hypertrichosis, hypertelorism, broad nasal bridge, and microcephaly (Iourov et al., 2012). The etiology of ID is principally genetic, with an allelic spectrum that includes highly penetrant single‐gene mutations, copy number variants (CNVs) of variable penetrance, and polygenic common variants individually of low penetrance. With more than 100 ID genes and loci identified so far, many more remain to be discovered (Cooper et al., 2015; Wieczorek, 2018).

Recent large‐scale genome sequencing initiatives of ID have identified a diverse set of genes that are predominantly impacted by de novo or rare, inherited, clinically relevant variants (single‐nucleotide variants [SNVs], indel, CNVs (Gilissen et al., 2014; Hu et al., 2016; Uddin, Sturge, Peddle, O'Rielly, & Rahman, 2011)). Mutations in these genes are often pleiotropic, and associated with a spectrum of neuropsychiatric and medical disorders. For example, mutations in *STXBP1* (OMIM 602926) and *CHD8* (OMIM 610528), and microdeletions in 15q13.3 and 22q11.2 are all reported to be characterized by a range of disorders, and such pleiotropy does seem to be typical of other genes implicated in brain disorders (Cheung et al., 2014; McCarthy et al., 2014; Uddin et al., 2017). A significant number of recessive candidate genes have also been identified in consanguineous families (Alazami et al., 2015).

Currently, chromosomal microarray (CMA) is the first‐tier diagnostic test, which can detect “clinically relevant” variants in 25% of individuals with neurodevelopmental disorders (including ID) defined as those variants that are pathogenic, likely pathogenic, or of unknown significance (variant of uncertain significance [VUS]) (Ho et al., 2016). The diagnostic yield from next‐generation sequencing (whole‐exome or genome sequencing) was recently reported to be 40%, making it a better choice for clinical diagnosis (Lavillaureix et al., 2017; Wright et al., 2018). Improvements in identifying smaller DNA structural variants, the contribution of mosaic mutations (Lim et al., 2017), and better characterization of VUS and intronic mutations will further increase the diagnostic yield.

Collective evidence from large‐scale independent studies has implicated more than 100 genes with ID (Hu et al., 2016, 2018). This large number of genes are involved in a diverse set of pathways and processes. These large number of genes and pathways, complicates genotype–phenotype relationship. Synaptic morphology has been implicated in numerous ID and neurodevelopmental disorder studies (Zoghbi & Bear, 2012), and deficits in dendritic arborization, pre‐ or postsynaptic density, and action potential propagation (and hence neurotransmitter release) have been shown to be associated with ID (Al Shehhi et al., 2018; Bhalla et al., 2008; Rump et al., 2016; Woodbury‐Smith et al., 2017). Additionally, genes that encode proteins which regulate cytoskeleton dynamics have been implicated in ID, including GTP/GDP‐bound state of RhoGTPases, such as *AP1S2* (OMIM 300629), *FGD1* (OMIM 300546*)*, and *SRGAP3* (OMIM 606525). Covalent modifications on DNA or posttranslational modifications on histones, known as epigenetic regulation, have been well associated with ID (Bowling et al., 2017).

In this study, we have applied whole‐genome microarray to investigate the genetic etiology of two siblings affected with ID and facial abnormalities.

## 2 MATERIALS AND METHODS

### 2.1 Ethical compliance and sample collection

The institutional ethical review committee (IERC) of Holy Family Red Crescent Medical College & Hospital, Dhaka, Bangladesh approved this study. We have analyzed a non‐consanguineous quartet family in which the two siblings have ID. Informed consent was obtained from the parents of the siblings in this study.

### 2.2 Clinical assessment

The two siblings were assessed at NeuroGen Technologies Ltd. Clinical evaluation included neurological and neurodevelopmental assessment by a neurologist and other clinicians. Formal parental interviews were undertaken and the siblings were examined. Information regarding their developmental history was collected, including medical history, history of infections, and dietary details. Physical examination, including morphometric assessment, was performed. More formal psychometric assessment of intellectual development was also carried out using the Wechsler Intelligence Scale for Children (WISC). Computerized tomography (CT) was also conducted on both siblings.

### 2.3 Whole‐genome microarray

We conducted genome‐wide microarray to identify chromosomal abnormalities (deletion/duplication/translocation and rearrangements) and investigated the changes in fluorescence intensities between the test specimen and the controls. Array comparative genomic hybridization (aCGH) chip technology was applied in Agilent system to detect chromosomal abnormalities. This microarray uses 33,000 probes spread across the genome to detect 372 genetic abnormalities (includes >60 loci in DECIPHER database reported for neurodevelopmental disorders) and also targets 41 subtelomeric regions that are vulnerable to chromosomal abnormalities. We have used rigorous multiple algorithmic techniques (MatLab and Java) and manual curation of the data to pinpoint genomic variation based on the normalized log2 intensities of the probes. Circular binary segmentation algorithm was used on the normalized –log2 values to detect CNVs. Our algorithm excludes all common CNVs found in our in‐house (NeuroGen) control population samples (9,610 samples) from analysis and only analyzed rare CNVs to infer their contribution to human diseases. Digestion, ligation, PCR, labeling, hybridization, and scanning were performed following standard protocols.

### 2.4 DNA extraction and qPCR experiment

DNA was extracted from peripheral blood sample using ReliaPrep™ Blood gDNA isolation kit (Promega) followed by the protocols provided by the manufacturer. The concentration and quality of DNA were measured using Nano Photometer C40 (Implen). Purified gDNA was quantified and diluted into 1 ng/µl to make sure that equal amounts of DNA in all wells and three replicates of reactions and NTC (no template control) were included. We identified two constraint genes (infrequent CNVs in normal population) within the duplication and deletion breakpoints to validate the chromosomal events. Two Taqman probes were ordered from ThermoFisher Scientific, *CNTNAP2* (NG_007092.3) (7q34‐q36.3 duplication), and *MEF2A* (NC_000015.10) (15q26.3 deletion). Taqman copy number reference assay RNase P (Part Number. 4403326, ThermoFisher Scientific) was also ordered. Taqman Universal Master Mix II with UNG (Part Number. 4440038, ThermoFisher Scientific) was used for the entire assay. Three different reaction mixes were prepared as follows for three assays. Taqman Universal Mater Mix II with UNG 10 µl, Taqman Copy number assay 20X 1 µl for each assays, Taqman Copy Number Reference Assay RNaseP 20X 1 µl, gDNA (1 ng/1 µl) 6 µl each, Nuclease free water 2 µl to a total of 20 µl reaction. Samples were prepared in a 96‐well plate in triplicates and performed the Standard Curve experiment the thermal profiles as 95°C for 10 min Hold, and to denature 95°C for 15 s, Anneal/Extend 60°C for 60 seconds for 40 cycles run. QuantStudio5 (ThermoFisher Scientific) qPCR machine was used to perform the experiment. After the run, the data were exported to text file and imported in Copy Caller Software Version 2.1 for the analysis of copy number variation data using Taqman Copy number assays.

### 2.5 Droplet digital PCR

ddPCR was carried out using QX 200 system (Bio‐Rad Laboratories, Inc.). The PCR mixtures–22 µl for each sample–were designed to assess copy number variation of *CNTNAP2* and *MEF2A* (TaqMan assay, Hs04975510_cn, and Hs05329359_cn, ThermoFisher), both genes were assayed on FAM channel, whereas the reference gene (TaqMan RNase P RPPH1, 14q11.2, ThermoFisher) was assayed on VIC channel. Reaction mixes contained 1X ddPCR Supermix (Bio‐Rad). Around 66 ng/reaction of DNA samples were added without enzymatic digestion. The PCR mixtures were partitioned using droplet generator, and then transferred into 96‐well PCR plate, the plate was run on the thermocycler following the manufacturer's protocol. Data analysis and acquisition were performed using QuantSoft software version 1.7.4.0917, which measures the number of positive and negative droplets. As a next step, the concentrations of the targets as copies/µl were determined by Poisson algorithm.

### 2.6 Candidate gene and pathway analysis

We applied multiple methods to identify candidate genes within the terminal duplication and deletion breakpoint. First, the “critical‐exon” method was applied, which identifies a set of constrained genes, that is, those that have exons with a low deleterious mutation burden in the population and high expression in the brain compared to other tissues (Uddin et al., 2016, 2014). Second, we identified genes that have been previously reported (from large‐scale sequencing cohorts) to carry de novo mutations (SNVs, Indels) or rare CNVs in multiple cases with broader neurodevelopmental disorders (i.e., ASD, ID). To obtain the brain expression level of the impacted gene, we analyzed 523 brain samples (prenatal to adult) RNA‐sequencing RPKM data (Kang et al., 2011). The candidate genes were analyzed for pathway enrichment using the Gene Ontology Panther classification tool (Mi et al., 2017). Well‐curated Gene Ontology pathways were analyzed for gene enrichment and a pathway was considered to be significant if the false discovery rate (FDR) threshold was <0.01.

## 3 RESULTS

### 3.1 Clinical phenotypes

**Figure 1:**
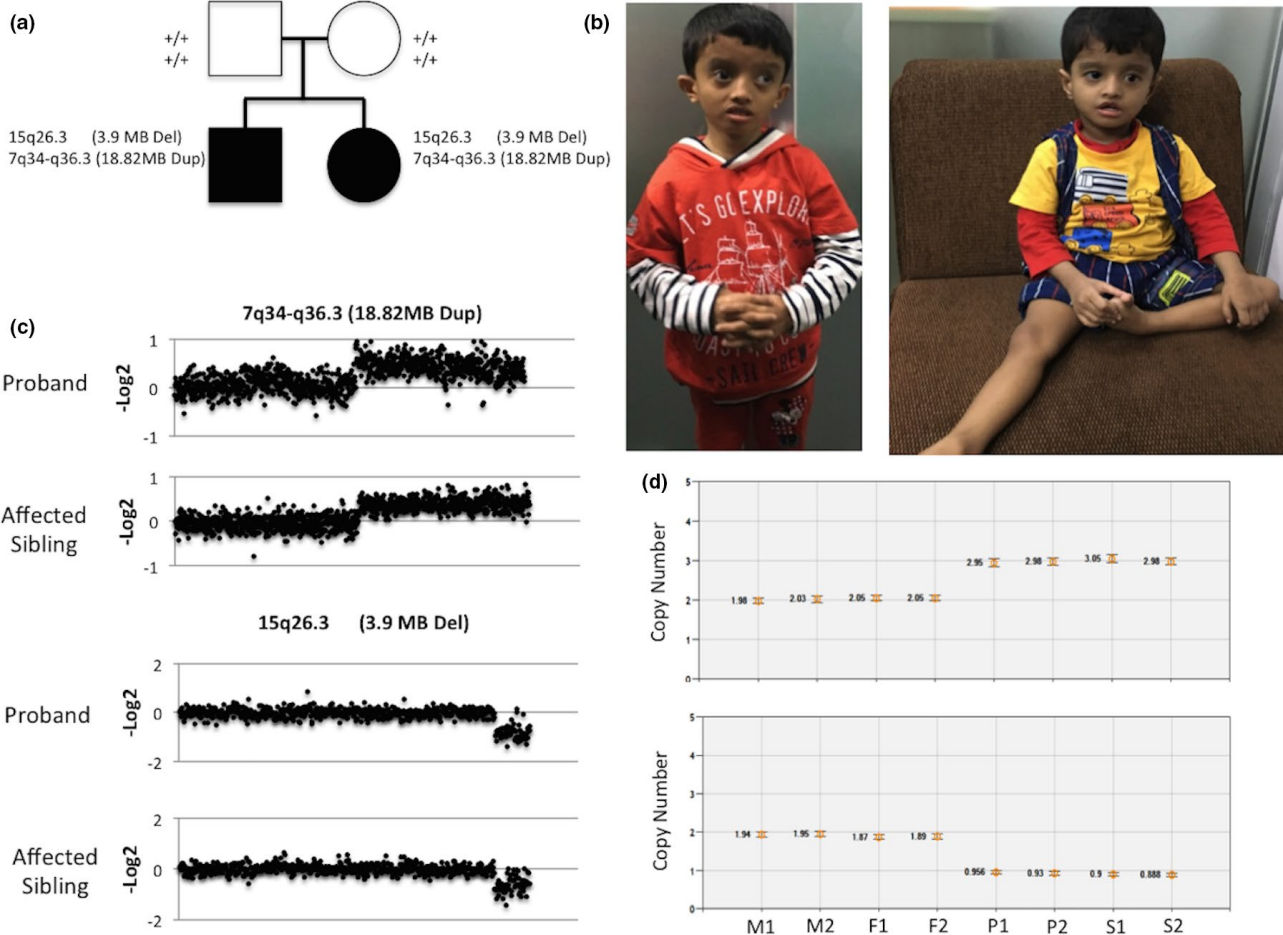
(a) The family pedigree with two affected siblings carrying the terminal de novo deletion and duplication. (b) Physical characteristic showing hypotonia and facial dysmorphism. (c) The probe intensities (−log2 ratio) of the 18.82 megabase (MB) subtelomeric duplication at 7q34‐q36.3 loci and the 3.9 MB deletion at 15q26.3 locus. (d) ddPCR results confirming de novo origin of the two variants. Two probes (targeting *MEF2A* and *CNTNAP2*) were used and tested in two technical replicates where label refers as F‐father, P‐proband, S‐affected sibling, M‐mother

Among the two siblings, the proband is a 7‐year‐old boy, who presented with global developmental delay and severely impaired language was diagnosed with a moderate ID (Full scale IQ: 50) (Figure 1a). He is the first born child to a non‐consanguineous parents with no family history of neurodevelopmental disorders. Following a normal, uncomplicated, and healthy pregnancy, he was delivered by lower uterine cesarean section (LUCS) following the identification of oligohydramnios. Hypotonia and subsequent delay in language and motor milestones were observed. He was not walking independently until the age of 6 years. There was no history of seizures, or any aggressive or other behavioral issues, although he exhibited sensory vulnerabilities. There was no evidence of ASD. Further examination revealed abnormal facial appearance with depressed nasal bridge, squint, hypertelorism, low set ear, and short stature (Figure 1b). His height, weight, and head circumference were 96 cm height‐for‐age Z score (HAZ = −4.90), 12 kg weight‐for‐age Z score (WAZ = −6.83), and 49 cm (Z‐score = −2.12), respectively. Vision, hearing, renal, gastro were normal and CT scan report was normal.

The affected sibling of the proband is a 4‐year‐old girl who also presented with global developmental delay with severely impaired language, and was diagnosed with ID. She did not co‐operate with IQ testing. She was full‐term delivered by LUCS following an uneventful pregnancy. She did not communicate verbally, and exhibited poor eye contact and nonverbal communication. She was hypotonic and did not crawl (Figure 1b). There was no history of seizures, and no behavioral abnormalities. There was no evidence of ASD. On examination, she had an abnormal facial appearance with low philtrum, squint, inverted v‐shaped thin upper lip, and microcephaly (occipitofrontal head circumference = 44 cm, Z‐score = −5.75). On examination, her height and weight were 86 cm (HAZ = −3.60) and 8.5 Kg (WAZ = −6.93), respectively. Vision and hearing were normal, and a CT scan was reported as normal.

No abnormalities were detected in GIT (gastrointestinal tract), renal, and cardiovascular system in both siblings.

### 3.2 Microarray analysis and validation

A pathogenic terminal duplication and deletion, with identical breakpoints, were detected in both siblings at 7q34‐q36.3 and 15q26.3 loci, respectively. The first variant is an 18.82 megabase (MB) duplication (Chr7: 140,518,420–159,345,972) impacting more than 100 genes. The second variant is a 3.90 MB deletion (chr15:98,085,693–101,991,189) impacting over 30 genes (Figure 1c). These CNVs impact the entire subtelomeric regions of chromosome 7 and 15. For validation, we applied qPCR probes targeting CNV constraint genes *CNTNAP2* and *MEF2A* in chromosome 7 and 15, respectively. The qPCR analysis showed (in all three replicates) that both variants are de novo in origin.

Based on the ddPCR, automatic thresholding determined the copy numbers, and it populated the droplets into four distinct groups. For some samples, the fluorescence amplitude threshold was set manually as a midpoint average. The average number of accepted droplets ranged from 9,825 to 11,618 droplets for the *MEF2A*, and from 8,972 to 16,053 droplets for the *CNTNAP2*. We used exclusion criteria on the basis of (a) total number of droplets <5,000 and (b) the fluorescence amplitude was significantly deviated from those of the same patients.

Both parents replica samples yielded copy number close to two (Figure 1d), which is the expected result for a normal diploid genome. On the other hand, ddPCR confirmed the copy number variation of both tested genes in both siblings which was in agreement with the qPCR results.

### 3.3 Candidate gene analysis

**Figure 2:**
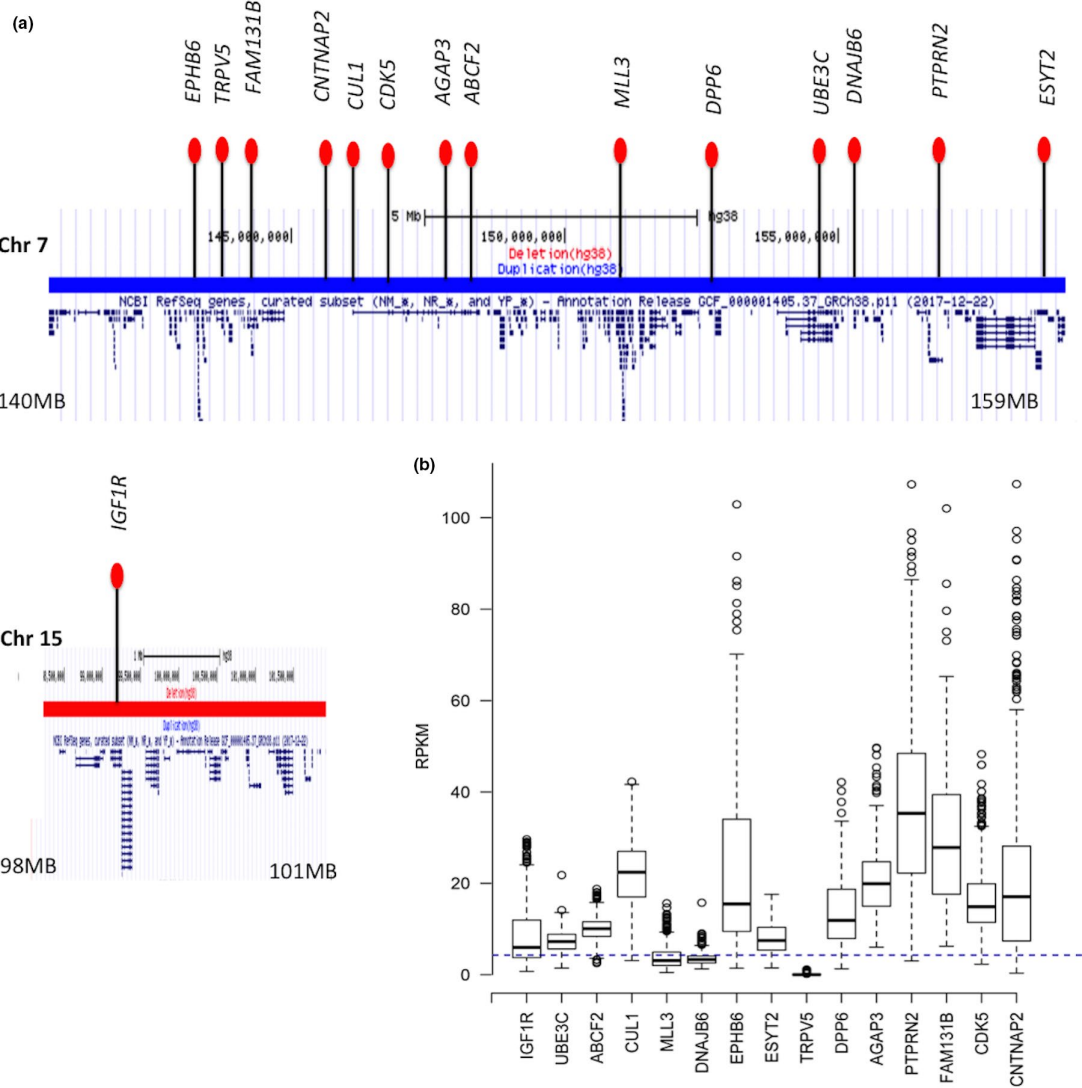
(a) The chromosomal location of the candidate genes within the deletion and duplication breakpoint. (b) The boxplot refers to human developmental brain expression (RPKM from RNA‐seq) level of 15 candidate genes. The blue line refers to the average expression level of all genes impacted by the two de novo copy number variants

Our Gene Ontology pathway enrichment analysis of the impacted genes within the CNV breakpoints identified “detection of chemical stimulus involved in sensory perception (GO:0050907)” and “nervous system process” (GO:0050877) pathways to be highly significant (FDR P < 2.1 × 10^–6^) and (FDR P < 9.0 × 10^–3^) after correction. Next, using “critical‐exon” analysis in conjunction with previously identified candidate genes, the following genes (from both loci) were considered potential candidates for the phenotypes observed in these siblings: *FAM131B, CNTNAP2, CUL1, CDK5, AGAP3, ABCF2*, *DPP6, DNAJB6,* and *PTPRN2* (Figure 2a). Next, we have identified *IGF1R, UBE3C, KMT2C, EPHB6, TRPV5, ESYT2, DPP6, PTPRN2,* and *CNTNAP2* reported to have de novo mutations in multiple (more than 2) independent cases with neurodevelopmental disorders, including ASD. The expression pattern (Figure 2b) of the candidate genes showed significantly high expression compared to all genes impacted by the two de novo CNVs.

## 4 DISCUSSION

Subtelomeric mutations are an infrequent cause of ID in approximately 0.5% of ID cases (Ruiter et al., 2007). The co‐occurrence of these two large terminal de novo CNVs in ID or broader developmental disorders cases is extremely rare. Moreover, the co‐occurrence of the two identical de novo events suggests either a gonadal mosaicism or a balanced translocation. Subtelomeric deletions of the 15q26.3 locus have been reported before as a causal variant for developmental disorders (O'Riordan et al., 2017). The 7q34‐q36.3 duplication, which affects more than 100 genes, might have also contribute to the phenotype, but less is known about the phenotypic consequences of duplications at this locus, particularly considering how large it is. Our Gene Ontology analysis suggests that these terminal CNVs are enriched with genes in the pathways for chemical stimulation, sensory perception, and nervous system processes.

We conducted an overlap analysis for the large duplication with known genomic disorders in mutation databases, and identified that it overlaps with variants previously implicated in Weaver syndrome, Noonan syndrome, Jeune syndrome, global developmental delay, and ASD. Previous reports of terminal duplications of various lengths within 7q have also been described in cases with severe to moderate facial dysmorphism and ID (Bartsch, Kalbe, Ngo, Lettau, & Schwinger, 1990; Morava et al., 2003; Romain et al., 1990; Speleman et al., 2000). It is therefore quite likely that this CNV is important in the pathogenesis of the phenotypes observed in these two siblings.

Both loci include multiple potential candidate genes, including *IGF1R, CNTNAP2,* and *DPP6*, all are well‐studied genes that are strongly associated with developmental and speech delays, congenital heart disease, epilepsy, diaphragmatic hernia, renal anomalies, and ID (Lin et al., 2013; Penagarikano et al., 2011; Yang et al., 2018). These candidate genes show significantly high expression in the developing human brain compared to other genes within the breakpoint (Figure 2b). Some of these other genes may be important for other phenotypes. For example, *SELENOS*, *SNRPA1*, and *PCSK6* may have a role in the development of the heart.

The clinical observation of both siblings identified similar and some unique features. The divot upper lip and low IQ score identifies the girl in severe spectrum of phenotypes compared to her brother. Her inability to crawl or talk at this age was marked as a difference compared to her affected brother. There can be multiple sources of risk factors that can explain the phenotypic heterogeneity between the siblings. Apart from these two high penetrant variants, genetic background, epigenetic factors, sex‐mediated factors, and environment also can influence the overall risk factor. Moreover, the relative roles of these two CNVs, and, specifically, whether a “two hit” mechanism is involved, are also unclear.

The phenotype for 15q26.3 subterminal deletions is highly heterogeneous and strongly associated with impaired prenatal and postnatal growth, developmental delay, dysmorphic features, and skeletal abnormalities. We could not find a recurrent breakpoint for the deletion in the DECIPHER database as well as in the literature. Similarly, there is no case in the literature with a recurrent or similar duplication breakpoint at 7q34‐q36.3 loci. Subtelomeric regions are more susceptible to aberrant rearrangements than other chromosomal regions (Brown et al., 2001; Saccone, De Sario, Della Valle, & Bernardi, 1992; Uddin et al., 2011). A large‐scale study carried out on submicroscopic subtelomeric aberrations in Chinese patients found that this chromosomal rearrangement impact 5.1% of children with clinically unexplained ID (Wu et al., 2010). Phenotypic manifestations usually depend on the number of genes involved (Helias‐Rodzewicz et al., 2002). Some common clinical feature may suggest various subtelomeric abnormalities including family history of ID, prenatal onset of growth retardation, postnatal growth abnormalities, at least two facial dysmorphic features, and at least one nonfacial dysmorphic feature and/or congenital abnormality (de Vries et al., 2001).

## 5 CONCLUSIONS

In this study, we have presented two siblings with two novel genomic rearrangements that are extremely rare. Both variants are de novo and high penetrant, impacting many important genes that are involved in neurodevelopmental processes. The co‐occurrence of more than one de novo event is extremely rare and suggestive of gonadal mosaicism. Although these variants are large and pathogenic, the phenotypes of the two siblings comprise similarities and differences. In order to validate our conclusion, we conducted ddPCR on the constraint genes and we demonstrated a normal diploid genomic DNA in parents. To unravel the mechanisms of genotype–phenotype differences between siblings with identical mutations, would require more detailed molecular experiments and modeling for epigenetic and other factors. Additionally, the detailed phenotype–genotype study of individual families, such as ours, will continue to be crucial in the identification of new, rare ID genes, and through the accumulation of evidence, the unique and shared patterns of phenotype will spurn new hypotheses concerning exact brain‐behavior mechanisms for these debilitating disorders.

## CONFLICT OF INTEREST

None declared.
